# *rac*-2-Methyl-3,4,5,6-tetra­hydro-2*H*-2,6-methano-1,3-benzoxazocin-4-one

**DOI:** 10.1107/S1600536810017848

**Published:** 2010-05-22

**Authors:** Viktor Kettmann, Jan Světlík, Lucia Veizerová

**Affiliations:** a Faculty of Pharmacy, Comenius University, Odbojarov 10, SK-83232 Bratislava, Slovakia

## Abstract

The title compound, C_12_H_13_NO_2_, represents a conformationally restricted 2-pyridone analogue of 1,4-dihydro­pyridine-type calcium antagonists and was selected for a crystal structure determination in order to explore some aspects of drug-receptor inter­action. In the mol­ecule, two stereogenic centres are of opposite chirality, whereas a racemate occurs in the crystal. It was found that the formally aminic N atom of the heterocycle is essentially *sp* ^2^-hybridized with the lone-pair electrons partially delocalized through conjugation with the adjacent carbonyl bond. As a result, the central pyridone ring assumes an unsymmetrical half-chair conformation. The critical 4-phenyl ring is fixed in a pseudo-axial and perpendicular orientation [dihedral angle 85.8 (1)°] with respect to the pyridone ring *via* an oxygen bridge. In the crystal a pair of centrosymmetric N—H⋯O hydrogen bonds connect mol­ecules of opposite chirality into a dimer. The dimers are packed by hydrophobic van der Waals inter­actions.

## Related literature

For background to 1,4-dihydro­pyridines (DHPs) as the most potent class of calcium-channel antagonists, see: Goldmann & Stoltefuss (1991 ▶); Kettmann *et al.* (1996 ▶). For bond-lengths in cyclic amino acids, see: Benedetti *et al.* (1983 ▶). For the preparation of the title compound, see: Světlík *et al.* (1990 ▶).
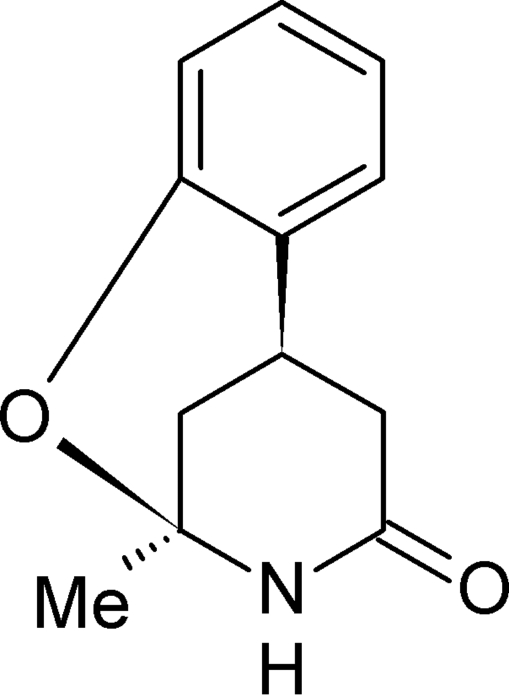


## Experimental

### 

#### Crystal data

C_12_H_13_NO_2_
*M* *_r_* = 203.23
Triclinic, 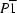

*a* = 5.564 (1) Å
*b* = 9.820 (2) Å
*c* = 10.596 (2) Å
α = 108.73 (1)°
β = 95.09 (2)°
γ = 103.60 (1)°
*V* = 524.37 (17) Å^3^
*Z* = 2
Mo *K*α radiation
μ = 0.09 mm^−1^
*T* = 296 K
0.30 × 0.25 × 0.20 mm

#### Data collection

Siemens P4 diffractometer
3821 measured reflections
2983 independent reflections
2242 reflections with *I* > 2σ(*I*)
*R*_int_ = 0.052
3 standard reflections every 97 reflections  intensity decay: none

#### Refinement

*R*[*F* ^2^ > 2σ(*F* ^2^)] = 0.049
*wR*(*F* ^2^) = 0.145
*S* = 1.03
2983 reflections
137 parameters
H-atom parameters constrained
Δρ_max_ = 0.26 e Å^−3^
Δρ_min_ = −0.17 e Å^−3^

### 

Data collection: *XSCANS* (Siemens, 1991 ▶); cell refinement: *XSCANS*; data reduction: *XSCANS*; program(s) used to solve structure: *SHELXS97* (Sheldrick, 2008 ▶); program(s) used to refine structure: *SHELXL97* (Sheldrick, 2008 ▶); molecular graphics: *PLATON* (Spek, 2009 ▶); software used to prepare material for publication: *SHELXL97*.

## Supplementary Material

Crystal structure: contains datablocks global, I. DOI: 10.1107/S1600536810017848/kp2259sup1.cif

Structure factors: contains datablocks I. DOI: 10.1107/S1600536810017848/kp2259Isup2.hkl

Additional supplementary materials:  crystallographic information; 3D view; checkCIF report

## Figures and Tables

**Table 1:** Hydrogen-bond geometry (Å, °)

*D*—H⋯*A*	*D*—H	H⋯*A*	*D*⋯*A*	*D*—H⋯*A*
N1—H1⋯O2^i^	0.86	2.07	2.9274 (15)	176

Symmetry code: (i) 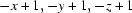.

**Fig. 1.**
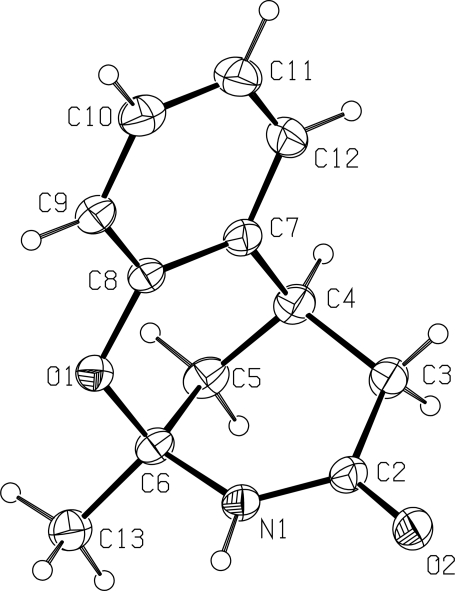
Displacement ellipsoid plot of (I) with the labelling scheme for the non-H atoms, which are drawn as 35% probability level.

## supplementary crystallographic information

### Comment

1,4-Dihydropyridines (DHPs) are known as the most potent class of calcium-channel antagonists widely used in clinical medicine. It was reported that the essential pharmacophore, recognizable by the DHP receptor, consists of the NH moiety, (substituted) phenyl ring and two ester groups (Goldmann & Stoltefuss, 1991). Nevertheless, we have previously observed that the rigid compound (I), lacking the ester groups in positions 3 and 5, retains some level of activity (Kettmann *et al.*, 1996). This implies that (I) presents its key pharmacophoric elements, *viz.* the NH and phenyl groups, in an optimal position and orientation for favourable binding to the complementary sites of the receptor. To establish the latter, a single-crystal X-ray analysis of (I) was undertaken.

The bond lengths and angles within the molecule (Fig. 1) are normal. As expected, there is a strong conjugation between N1 and the C2=O2 carbonyl bond, as usually observed for cyclic amino acids (Benedetti *et al.*, 1983).

As mentioned above, the main aim of this work was to determine the three-dimensional disposition of the key pharmacophoric groups, i.e. the phenyl and NH moieties (Fig. 1). The conformation of the central heterocycle acts as a scaffold to orient substituents in space. Thus, the pyridone ring adopts an unsymmetrical half-chair conformation in which atoms C6, N1, C2 and C3 are coplanar with r.m.s. deviation of 0.012 (1) Å, and atoms C4 and C5 are displaced from this plane by -0.348 (3) and 0.470 (3) Å, respectively. The phenyl ring at C4 occupies a pseudoaxial position (Fig. 1) and is fixed approximately in a perpendicular orientation with respect to the mean plane of the pyridone ring [dihedral angle 85.8 (1)°]; the ring is rotated on the C4—C7 bond in such a manner that it almost eclipses the C4—C5 bond [dihedral angle C5—C4—C7—C8 23.0 (2)°].

The crystal packing is governed by an intermolecular hydrogen bond *N*—*H*···*O*(carbonyl) (Table 1); as a result, the molecules associate into pairs to form hydrogen-bonded dimers across the centre of symmetry at (1/2,1/2,1/2). The dimers are packed by van der Waals forces only.

### Experimental

As described in details earlier (Světlík *et al.*, 1990), the title compound, (I), was prepared by cyclocondensation of 4-(2-hydroxyphenyl)but-3-en-2-one with Meldrum's acid in refluxing ethanol for 4 hours (27% yield; m.p. 530-531 K). Single crystals suitable for an X-ray analysis were obtained by slow crystallization of ethanol solution.

### Refinement

H atoms were visible in difference maps and were subsequently treated as riding atoms with distances N—H = 0.86, C—H 0.93 (CH_arom_), 0.97 (CH_2_) or 0.98 (CH) and 0.96 Å (CH_3_); U_iso_ of the H atoms were set to 1.2 (1.5 for the methyl H atoms) times U_eq_ of the parent atom.

### Crystal data

**Table:**

C_12_H_13_NO_2_	*Z* = 2
*M**_r_* = 203.23	*F*(000) = 216
Triclinic, *P*1	*D*_x_ = 1.287 Mg m^−^^3^
Hall symbol: -P 1	Melting point: 530 K
*a* = 5.564 (1) Å	Mo *K*α radiation, λ = 0.71073 Å
*b* = 9.820 (2) Å	Cell parameters from 20 reflections
*c* = 10.596 (2) Å	θ = 7–18°
α = 108.73 (1)°	µ = 0.09 mm^−^^1^
β = 95.09 (2)°	*T* = 296 K
γ = 103.60 (1)°	Prism, colourless
*V* = 524.37 (17) Å^3^	0.30 × 0.25 × 0.20 mm

### Data collection

**Table:**

Siemens P4 diffractometer	*R*_int_ = 0.052
Radiation source: fine-focus sealed tube	θ_max_ = 30.0°, θ_min_ = 2.1°
graphite	*h* = −1→7
ω/2θ scans	*k* = −12→12
3821 measured reflections	*l* = −14→14
2983 independent reflections	3 standard reflections every 97 reflections
2242 reflections with *I* > 2σ(*I*)	intensity decay: none

### Refinement

**Table:**

Refinement on *F*^2^	Primary atom site location: structure-invariant direct methods
Least-squares matrix: full	Secondary atom site location: difference Fourier map
*R*[*F*^2^ > 2σ(*F*^2^)] = 0.049	Hydrogen site location: inferred from neighbouring sites
*wR*(*F*^2^) = 0.145	H-atom parameters constrained
*S* = 1.03	*w* = 1/[σ^2^(*F*_o_^2^) + (0.0608*P*)^2^ + 0.0887*P*] where *P* = (*F*_o_^2^ + 2*F*_c_^2^)/3
2983 reflections	(Δ/σ)_max_ = 0.001
137 parameters	Δρ_max_ = 0.26 e Å^−^^3^
0 restraints	Δρ_min_ = −0.17 e Å^−^^3^

### Special details

**Table:**

Geometry. All esds (except the esd in the dihedral angle between two l.s. planes) are estimated using the full covariance matrix. The cell esds are taken into account individually in the estimation of esds in distances, angles and torsion angles; correlations between esds in cell parameters are only used when they are defined by crystal symmetry. An approximate (isotropic) treatment of cell esds is used for estimating esds involving l.s. planes.
Refinement. Refinement of *F*^2^ against ALL reflections. The weighted *R*-factor wR and goodness of fit *S* are based on *F*^2^, conventional *R*-factors *R* are based on *F*, with *F* set to zero for negative *F*^2^. The threshold expression of *F*^2^ > σ(*F*^2^) is used only for calculating *R*-factors(gt) etc. and is not relevant to the choice of reflections for refinement. *R*-factors based on *F*^2^ are statistically about twice as large as those based on *F*, and *R*- factors based on ALL data will be even larger.

### Fractional atomic coordinates and isotropic or equivalent isotropic displacement parameters (Å^2^)

**Table:**

	*x*	*y*	*z*	*U*_iso_*/*U*_eq_	
N1	0.7300 (2)	0.47999 (13)	0.63412 (11)	0.0368 (3)	
H1	0.6390	0.5366	0.6239	0.044*	
C2	0.7192 (3)	0.36033 (16)	0.52401 (13)	0.0390 (3)	
C3	0.8893 (3)	0.26336 (19)	0.53502 (14)	0.0464 (3)	
H3A	1.0279	0.2843	0.4883	0.056*	
H3B	0.7957	0.1590	0.4900	0.056*	
C4	0.9955 (3)	0.28856 (17)	0.68250 (14)	0.0431 (3)	
H4	1.1317	0.2410	0.6836	0.052*	
C5	1.0990 (2)	0.45662 (18)	0.75980 (15)	0.0432 (3)	
H5A	1.1854	0.4750	0.8498	0.052*	
H5B	1.2176	0.5015	0.7127	0.052*	
C6	0.8791 (2)	0.52435 (15)	0.76906 (12)	0.0353 (3)	
C7	0.7943 (3)	0.22442 (16)	0.75190 (13)	0.0391 (3)	
C8	0.6692 (2)	0.31756 (14)	0.83327 (12)	0.0345 (3)	
C9	0.4875 (3)	0.26224 (16)	0.90078 (14)	0.0405 (3)	
H9	0.4077	0.3257	0.9555	0.049*	
C10	0.4269 (3)	0.11180 (18)	0.88560 (16)	0.0506 (4)	
H10	0.3057	0.0745	0.9303	0.061*	
C11	0.5454 (4)	0.01657 (18)	0.80448 (17)	0.0570 (4)	
H11	0.5035	−0.0843	0.7942	0.068*	
C12	0.7267 (3)	0.07306 (18)	0.73887 (16)	0.0516 (4)	
H12	0.8059	0.0088	0.6847	0.062*	
C13	0.9524 (3)	0.69372 (17)	0.83480 (15)	0.0470 (3)	
H13A	1.0388	0.7229	0.9260	0.071*	
H13B	0.8040	0.7276	0.8358	0.071*	
H13C	1.0608	0.7377	0.7842	0.071*	
O1	0.72022 (17)	0.46921 (10)	0.85435 (9)	0.0372 (2)	
O2	0.5800 (2)	0.33318 (13)	0.41568 (10)	0.0537 (3)	

### Atomic displacement parameters (Å^2^)

**Table:**

	*U*^11^	*U*^22^	*U*^33^	*U*^12^	*U*^13^	*U*^23^
N1	0.0382 (6)	0.0428 (6)	0.0337 (5)	0.0192 (5)	0.0020 (4)	0.0147 (4)
C2	0.0442 (7)	0.0449 (7)	0.0338 (6)	0.0194 (6)	0.0061 (5)	0.0170 (5)
C3	0.0549 (8)	0.0582 (9)	0.0383 (7)	0.0329 (7)	0.0129 (6)	0.0197 (6)
C4	0.0413 (7)	0.0589 (9)	0.0421 (7)	0.0304 (6)	0.0100 (5)	0.0228 (6)
C5	0.0308 (6)	0.0599 (9)	0.0457 (7)	0.0161 (6)	0.0043 (5)	0.0257 (6)
C6	0.0323 (6)	0.0418 (7)	0.0331 (6)	0.0104 (5)	0.0020 (5)	0.0160 (5)
C7	0.0433 (7)	0.0459 (7)	0.0332 (6)	0.0208 (6)	0.0016 (5)	0.0158 (5)
C8	0.0362 (6)	0.0375 (6)	0.0311 (6)	0.0132 (5)	−0.0004 (5)	0.0135 (5)
C9	0.0405 (7)	0.0459 (7)	0.0373 (6)	0.0129 (6)	0.0038 (5)	0.0178 (5)
C10	0.0515 (8)	0.0509 (9)	0.0499 (8)	0.0075 (7)	0.0049 (6)	0.0245 (7)
C11	0.0732 (11)	0.0395 (8)	0.0568 (9)	0.0123 (7)	0.0033 (8)	0.0199 (7)
C12	0.0673 (10)	0.0449 (8)	0.0459 (8)	0.0275 (7)	0.0051 (7)	0.0135 (6)
C13	0.0503 (8)	0.0423 (7)	0.0434 (7)	0.0061 (6)	0.0000 (6)	0.0154 (6)
O1	0.0421 (5)	0.0377 (5)	0.0367 (5)	0.0154 (4)	0.0102 (4)	0.0156 (4)
O2	0.0705 (7)	0.0545 (6)	0.0361 (5)	0.0309 (5)	−0.0060 (5)	0.0104 (4)

### Geometric parameters (Å, °)

**Table:**

N1—C2	1.3489 (17)	C6—C13	1.518 (2)
N1—C6	1.4634 (16)	C7—C12	1.402 (2)
N1—H1	0.8600	C7—C8	1.4011 (18)
C2—O2	1.2399 (16)	C8—O1	1.3873 (16)
C2—C3	1.5137 (19)	C8—C9	1.3959 (19)
C3—C4	1.5399 (19)	C9—C10	1.388 (2)
C3—H3A	0.9700	C9—H9	0.9300
C3—H3B	0.9700	C10—C11	1.386 (2)
C4—C7	1.518 (2)	C10—H10	0.9300
C4—C5	1.526 (2)	C11—C12	1.385 (3)
C4—H4	0.9800	C11—H11	0.9300
C5—C6	1.5203 (18)	C12—H12	0.9300
C5—H5A	0.9700	C13—H13A	0.9600
C5—H5B	0.9700	C13—H13B	0.9600
C6—O1	1.4568 (16)	C13—H13C	0.9600
			
C2—N1—C6	127.38 (11)	N1—C6—C5	109.98 (11)
C2—N1—H1	116.3	C13—C6—C5	114.61 (12)
C6—N1—H1	116.3	C12—C7—C8	117.50 (14)
O2—C2—N1	121.03 (12)	C12—C7—C4	122.47 (13)
O2—C2—C3	120.90 (12)	C8—C7—C4	120.03 (12)
N1—C2—C3	118.03 (12)	O1—C8—C9	115.93 (11)
C2—C3—C4	113.10 (11)	O1—C8—C7	122.84 (12)
C2—C3—H3A	109.0	C9—C8—C7	121.22 (13)
C4—C3—H3A	109.0	C10—C9—C8	119.44 (14)
C2—C3—H3B	109.0	C10—C9—H9	120.3
C4—C3—H3B	109.0	C8—C9—H9	120.3
H3A—C3—H3B	107.8	C11—C10—C9	120.64 (15)
C7—C4—C5	108.71 (11)	C11—C10—H10	119.7
C7—C4—C3	111.56 (12)	C9—C10—H10	119.7
C5—C4—C3	108.61 (12)	C12—C11—C10	119.36 (15)
C7—C4—H4	109.3	C12—C11—H11	120.3
C5—C4—H4	109.3	C10—C11—H11	120.3
C3—C4—H4	109.3	C11—C12—C7	121.84 (15)
C6—C5—C4	107.95 (11)	C11—C12—H12	119.1
C6—C5—H5A	110.1	C7—C12—H12	119.1
C4—C5—H5A	110.1	C6—C13—H13A	109.5
C6—C5—H5B	110.1	C6—C13—H13B	109.5
C4—C5—H5B	110.1	H13A—C13—H13B	109.5
H5A—C5—H5B	108.4	C6—C13—H13C	109.5
O1—C6—N1	108.80 (10)	H13A—C13—H13C	109.5
O1—C6—C13	105.03 (11)	H13B—C13—H13C	109.5
N1—C6—C13	109.14 (11)	C8—O1—C6	116.70 (10)
O1—C6—C5	109.04 (10)		
			
C6—N1—C2—O2	178.31 (13)	C3—C4—C7—C8	−96.72 (15)
C6—N1—C2—C3	−3.9 (2)	C12—C7—C8—O1	179.75 (11)
O2—C2—C3—C4	−165.37 (14)	C4—C7—C8—O1	0.11 (18)
N1—C2—C3—C4	16.9 (2)	C12—C7—C8—C9	1.13 (19)
C2—C3—C4—C7	71.70 (16)	C4—C7—C8—C9	−178.51 (11)
C2—C3—C4—C5	−48.10 (17)	O1—C8—C9—C10	−179.60 (11)
C7—C4—C5—C6	−54.82 (14)	C7—C8—C9—C10	−0.89 (19)
C3—C4—C5—C6	66.74 (14)	C8—C9—C10—C11	0.1 (2)
C2—N1—C6—O1	−97.04 (15)	C9—C10—C11—C12	0.4 (2)
C2—N1—C6—C13	148.87 (14)	C10—C11—C12—C7	−0.1 (2)
C2—N1—C6—C5	22.34 (18)	C8—C7—C12—C11	−0.6 (2)
C4—C5—C6—O1	66.77 (13)	C4—C7—C12—C11	178.99 (13)
C4—C5—C6—N1	−52.46 (14)	C9—C8—O1—C6	−170.69 (10)
C4—C5—C6—C13	−175.85 (11)	C7—C8—O1—C6	10.63 (16)
C5—C4—C7—C12	−156.59 (12)	N1—C6—O1—C8	76.17 (13)
C3—C4—C7—C12	83.67 (16)	C13—C6—O1—C8	−167.08 (10)
C5—C4—C7—C8	23.03 (16)	C5—C6—O1—C8	−43.79 (14)

### Hydrogen-bond geometry (Å, °)

**Table:**

*D*—H···*A*	*D*—H	H···*A*	*D*···*A*	*D*—H···*A*
N1—H1···O2^i^	0.86	2.07	2.9274 (15)	176

Symmetry codes: (i) −*x*+1, −*y*+1, −*z*+1.
